# Functional Aphonia

**Published:** 1908-04-25

**Authors:** 


### Laryngology and Rhinology.

### FUNCTIONAL APHONIA.

Functional aphonia is the result of paralysis, or, strictly speaking, paresis of the adductor muscles of the cords, and has an ætiology entirely different from that of abductor paralysis. It has been thoroughly established that any organic lesion of the path of innervation of the laryngeal muscles, from the bulbar nuclei downwards, constantly produces paralysis of the abductor muscles in the first instance, provided that it acts slowly and progressively. When the nerve-path is completely destroyed all the muscles are paralysed and the cord assumes the cadaveric position. Therefore, an organic lesion cannot cause an isolated paralysis of any of the adductor muscles. The larynx may be considered to have two somewhat antagonistic functions: that of respiration subserved by the abductors, and that of phonation subserved by the adductors. The former is more ancient and vital, and can therefore be maintained by the bulbar centres without the assistance of the cerebral cortex, while the phonatory function is more directly controlled by the will, and possesses a well-marked cortical centre in each hemisphere. Stimulation of either cortical centre produces adduction of both cords, from which it appears that each cord is controlled from both cerebral hemispheres; so that a unilateral lesion above the bulbar nuclei should not cause any paralysis, and in point of fact the laryngeal muscles are unaffected in cases of hemiplegia. A bilateral cortical lesion would be expected to result in adductor paralysis of both cords, and this is exactly what has been found to occur clinically, though such cases are, of course, of extreme rarity.

As the phonatory function involves the very fine adjustment of a delicate piece of mechanism, and is in close association with the higher cortical centres, it is but natural that it should readily be thrown out of gear by any functional disturbance. Functional aphonia is thus a common manifestation of hysteria, but adductor paresis is also due to local disease, that is to inflammation, infiltration, or degeneration of the muscles, which interferes directly with their action. Indeed, it should be clearly borne in mind that this affection is, in the majority of cases, not purely hysterical, but is characteristic rather of a general loss of tone, in which any local laryngeal condition may predispose to functional aphonia. Thus some patients completely lose the voice during a slight attack of laryngitis. The affection is especially common in consumptives owing to the combination of debility with frequent cough and a tendency to laryngitis; so much is this the case that in obstinate cases the possibility of phthisis should always be remembered. The affection occurs in men as well as in women, though far less frequently.

The aphonia may be complete, or the voice may be brought out by special effort. In purely hysterical cases the cough is usually not aphonic. The onset is sudden, as is also the recovery, and the voice, when it returns, is not hoarse. In the most marked hysterical cases the power of whispering may be lost as well, "hysterical mutism," and rarely there is inability to whisper while phonation is normal, a condition to which the term "apsythyria" has been applied. Occasionally the patient may be able to sing, although unable to speak above a whisper. In cases where the neurotic element is less marked, and where the aphonia complicates laryngitis, the onset and recovery are less rapid, and hoarseness alternates with the aphonia.

The paralysis is always bilateral; it is hardly ever complete, and usually amounts only to imperfect apposition of the cords. There are two pairs of adductor muscles and one unpaired muscle, the arytenoideus, which draws the arytenoids together and closes the cartilaginous glottis; the crico-arytenoidei laterales rotate the vocal processes inwards, and thus close the ligamentous glottis; the thyro-arytenoidei make straight and taut the edges of the vocal cords. The adductor muscles may all fail together, when the cords remain at a variable distance from the middle line on attempted phonation. Sometimes the arytenoideus alone remains active, in which case the arytenoid cartilages approach posteriorly, but the vocal processes and cords remain divergent. Frequently the thyro-arytenoidei are alone affected, leaving an elliptical gap between the cords while the cartilaginous glottis is well closed; and rarely the arytenoideus is the only muscle paralysed when the cords and vocal processes come together, but a triangular space is left between the arytenoids.

It is possible to mistake bilateral total paralysis for adductor paralysis, for in both cases the cords fail to come together on phonation. Inspection during deep inspiration will decide the question, for in the former affection the cords then remain motionless, while in adductor paralysis they become more widely separated.

Cases associated with laryngeal catarrh require to be treated as for the latter affection; stimulating local applications from an atomiser or a steam-inhaler are usually indicated. Where the condition is purely a neurosis, local stimulation of the larynx is to be employed. Any form of stimulation may prove successful, such as the local application of an astringent solution on a laryngeal applicator, or electricity applied externally to the neck, and the application of high-frequency currents has been known to give the same result. But it is very important not to allow the larynx to become accustomed to mild or inefficient stimuli, for every ineffectual application makes the case more intractable. The general health should therefore be attended to and strychnine administered in full doses before any local treatment is undertaken; for the latter only faradisation with a laryngeal electrode is to be recommended, and the current should be from the first strong enough to be decidedly painful. The patient should be persuaded to speak immediately after the application. Intractable cases frequently relapse, and in these attention should be paid to correct voice production, for the majority of such patients use faulty methods of speaking.

